# Lost in translation: a case-study of the travel of lean thinking in a hospital

**DOI:** 10.1186/s12913-015-1081-z

**Published:** 2015-09-21

**Authors:** Hege Andersen, Kjell Arne Røvik

**Affiliations:** 1 University Hospital of North Norway, Box 100, 9038 Tromsø, Norway; 2 Department of Sociology, Political Science, and Community Planning, Faculty of Humanities, Social Sciences, and Education, University of Tromsø, Hansine Hansens v 14, 1919 Tromsø, Norway

**Keywords:** Quality improvement, Lean thinking, Healthcare, Context, Implementation, Translation, Hospital, Norway

## Abstract

**Background:**

Lean thinking as a quality improvement approach is introduced in hospitals worldwide, although evidence for its impact is scarce. Lean initiatives are social, complex and context-dependent. This calls for a shift from cause–effect to conditional attributions to understand how lean works. In this study, we bring attention to the transformative power of local translation, which creates different versions of lean in different contexts, and thereby affect the evidence for lean as well as the success of lean initiatives within and among hospitals.

**Methods:**

We explored the travel of lean within a hospital in Norway by identifying local actors’ perceptions of lean through their images of enablers for successful interventions. These attributions describe the characteristics of lean in use, i.e. the prevailing version of lean. Local actors’ perceptions of enablers for lean interventions were collected through focus group interviews with three groups of stakeholders: managers, internal consultants and staff. A questionnaire was used to reveal the enablers relative importance.

**Results:**

The enablers known from the literature were retrieved at the case hospital. The only exception was that external expert change agents were not believed to promote lean. In addition, the stakeholders added a number of new and supplementary enablers. Two-thirds of the most important enablers for success were novel, local ones. Among these were a problem, not method focus, a bottom-up approach, the need of internal consultants, credibility, realism and patience. The local actors told different stories about local enablers and had different images of lean depending on their hierarchical level.

**Discussion:**

By comparing and analyzing the findings from the literature review, the focus groups and the survey, we deduced that the travel of lean within the hospital was affected by *three principles of translation*: the practical, the pragmatic, and the sceptical. Further, *three logics of translation* were in play: translation as a funnel, a conscious sell-in, and a wash-out. This resulted in various local versions of lean.

**Conclusions:**

We conclude that lean, introduced by the management, communicated by the internal consultants, and used by the staff, is transformed more than once within the hospital. Translation is part of the explanation for the lack of evidence for lean, and translation can be decisive for outcomes.

**Electronic supplementary material:**

The online version of this article (doi:10.1186/s12913-015-1081-z) contains supplementary material, which is available to authorized users.

## Background

Quality improvement by the use of *lean thinking* is introduced in many hospitals worldwide [1]. Lean thinking is a systematic quality improvement approach to identify and eliminate non-value-adding activities in work processes [2]. It is argued that lean’s focus on zero defects, no delay, continuous improvement and *just in time* make lean especially suited for healthcare [3, 4]. However, in practice, lean interventions are characterized by high variance; that is, high heterogeneity of the context and the intervention itself - the content, the application and the outcomes of lean [5–9]. Studies that apply an experimental design have trouble finding significant effects of lean, and qualitative studies showing positive effects are characterized by a narrow application and limited organisational reach [6, 10–15]. In sum, there is a lack of evidence for lean impact in healthcare.

A recent review of enablers for successful lean interventions conclude that more attention should be paid to local application and translation [16], echoing other studies that assert the importance of context to understand variations in outcomes of such interventions [6, 17–19]. The common argument for a context approach is that outcomes vary because contexts are different. Thus, one must map contexts to understand why similar interventions produce different outcomes. Our approach is complementary, maintaining that actors in different contexts translate and transform a lean intervention differently, thereby creating different versions of lean, and thus, different interventions in different contexts. To understand variations in outcomes of lean interventions, one must also understand why and how the intervention itself is changed. This implies a shift from cause–effect to conditional attributions and to the transformative power of local translation processes [20–22].

The aim of this study is to explore the travel of lean into and within one case hospital in Norway by emphasizing how local interpretation at three different hierarchical levels at the hospital leads to the emergence of various versions of lean. Our two main research questions are:

- Is lean, defined by enablers for lean, translated during its travel within the hospital? If so, where do the translations take place, and who are the translators?
- How is lean translated? Do such translation processes have any rules or regularities?

The answers to the two research questions may contribute to suggest to what extent variations in outcomes can be considered a consequence of how lean interventions are translated.

## Methods

This study’s empirical basis is a lean initiative at a university hospital in Norway. The hospital underwent a complex merger and restructuring process between 2007 and 2010 [23], during which lean was introduced as an enterprise-wide program intended to improve patient pathways, including quality of care and work conditions, and increase hospital efficiency. The lean techniques used included value stream mapping of work processes, identification and elimination of activities that did not add value, maintaining value-adding activities in work processes running without any delay [24, 25].

The hospital chose a strict approach to implementing lean, using trained internal lean consultants, standardized schemes and routines. The standards were anticipated to prevent comprehensive variations among the different interventions across the hospital. However, an evaluation after five years of lean experience documented that the lean impact, where improved standards were adopted, routinised, integrated, and the intended effects accomplished, varied considerably among the lean initiatives at the hospital [26].

### Data collection

Enablers for lean interventions were identified by a systematic review of literature reviews concerning lean in hospitals (2000–2012) [16]. Local enablers for lean interventions were collected through separate, semi-structured focus group interviews with three groups of stakeholders: leaders of lean steering-groups (heads of divisions), internal lean consultants, and staff participating in redesigning patient pathways. All the participants had detailed first-hand experience of lean projects and processes.

Participation in the focus groups was restricted to employees involved in lean interventions implemented in the period from 2008 to 2012. The focus groups were considered to be a representative sample of relatively small populations; 8 of 10 steering-group leaders and 14 of 17 internal consultants attended. The 11 members of improvement groups that attended were collected from a list including all 258 former members. Two participants from each of the 17 projects were invited by drawing lots. The sample closure at 11 was reasoned by a judgment of a sufficient, representative sample size.

The focus group interviews were conducted in March 2013. Each interview lasted 2–3 h, and was taped and transcribed by the corresponding author, in consultation with the co-author. Both authors were running the focus groups. The critical incident technique (CIT) was used during the data collection, with emphasis placed on the incidents that had made the most significant contributions to the improvement activity [27]. The participants were asked to identify the two to three most important incidents that contributed to the lean project’s success. Each incident was only registered once, even if it was mentioned several times. The participants were not briefed on the enablers identified by the literature review [16].

All the enablers were assigned to larger categories by using work-sheets to secure a systematic classification of data [28]. The classification was carried out to develop a more specific and practically focused state of knowledge. The analytical approach of developing broad conclusions was believed to increase the relevance of the study results [29]. After examining all the reported enablers and grouping them with similar ones, we ended up with a list comprising 44 enablers. They were systematized according to which domain of the intervention they touched upon: *context* covered the setting in which the intervention is deployed, *content* referred to the characteristics of the intervention itself, *application* related to the process through which the intervention was implemented, and *outcome* covered the results and maintenance phase after implementation [6].

To ensure that all relevant factors were identified, the respondents had an opportunity to launch additional enablers by e-mail two weeks after the interview. This to secure that the final list included all the enablers that the stakeholders believed was important to successful lean interventions.

In order to reveal the relative importance of the enablers, an electronic questionnaire was mailed to 363 registered former participants in lean projects, during the period from April to May, 2013. We used *Quest-back* in order to ensure the anonymity of the respondents. The questionnaire contained a list of the locally identified enablers. The respondents were asked to point out the three most important enablers for quality improvement concerning the setting, content, local application and outcomes of lean.

A total of 195 people completed the questionnaire, of which 165 were included in the survey, leaving out 30 that reported that they had no lean experience, as they never attended the lean project they were invited to. The remaining sample was organisationally and professionally representative, regarding the three hierarchical groups that constituted the population. Characteristics of the participants in the focus groups and the survey are given in additional files (Additional file 1).

Approval was obtained from the Data Protection Official for Research (PVO), who confirmed that a more comprehensive ethical approval or informed consent was not necessary.

## Results

The enablers identified in the literature review were also reported at the hospital. The only exception was that external expert change agents, networks and sponsorships [30, 31] was not believed to trigger change by the stakeholders at the case hospital. However, the focus groups added a number of new and supplementary enablers not identified in the literature review.

Table 1 presents the enablers identified at the case hospital. They are organized according to whether they are retrieved from the literature review or novel, local ones. A further description of the local enablers is presented as an additional file (Additional file 2).

**Table 1:** Local enablers for lean improvement

Part of intervention	Context	Content	Application	Outcomes
	Situation and organisation	Characteristics of the intervention	Local delivery process	Results and maintenance
Reviewed pre-conditions	Experience	Adaption	Teamwork	Supportive culture
	Belief	Customer focus	Administrative support	Communication
	IT-systems	Training	Physicians	Holistic approach
	Competence	Resources	Management	Continuous improvement
	Alignment	Accurate data	Staff involvement	Measurement
	Vision			System-wide scope
	External support^a^			
Local pre-conditions	Preparation	Bottom-up	Credibility	Compatible to professional values
	Need for change	Dedication to lean	Internal consultants	Data feedback
	Anchoring in management, department or staff	Process orientation	Group composition	Smooth transition
	Management structure support	Priority setting tool	Operational	Realism and patience
		Visual and simple, less resource demanding	Sufficient participation	Few, palpable measures
			Problem, not method focus	Follow-up structure

^a^Enabler only identified in the review

The findings from the survey contribute to knowledge of the enablers’ relative contribution to lean success. Table 2 presents the 12 most important enablers according to the stakeholders at the case hospital.

**Table 2:** The most important enablers for change, results from the questionnaire (*n* = 165)

Context	Content	Application	Outcomes
**Management structure support**	Customer focus	Team work	**Few, palpable measures**
**Organisational structural support, coordination and continuity**	Include patient and workforce value creation and improvements	Multi-skilled and multi-disciplinary team collaboration including decision-making	**Concrete, quick results and visual success-stories**
Vision	**Bottom-up**	**Credibility**	**Realism and patience**
Targets of urgency and direction, but realistic, simple and practical solutions	**Improvement suggestions from floor, voluntariness due to initiative**	**No bragging, trustworthiness, no camouflaged dismissals or cuts**	**Distinct mandate, demarcation, smaller projects, adjustments possible**
**Need for change**	**Problem, not method focus**	**Internal consultants**	Holistic approach
**Perceived need, potential for improvement**	**Lean as a meeting place**	**Project management skills, mentors and network**	Lean as a entire value system, embracing every day improvement

Bold: locally identified enablers

Approximately half of the reviewed enablers were shared as important by the management, the consultants and the staff. Most of these related to the content of lean and the local application. When separated among the three groups, the preferred enablers and their relative importance diverged. The main results distributed across the three hierarchical levels are presented in the following paragraphs and detailed in additional files (Additional files 3 and 4).

### Management

In the management focus group, many of the reviewed enablers were shared. Exceptions, that is, enablers not mentioned, were belief in benefits as motivating, communication and feedback to staff, and a holistic lean approach. Among local enablers, the management did not launch a need for credibility or goals compatible to professional values. These findings are also reflected in the survey, as the management did not identify credibility or realism and patience as being among the most important enablers. They gave preference to their own role as management and the need for a smooth transition from project to everyday work.

### Internal consultants

The internal consultants recognized all of the reviewed enablers except a need for prior experience in quality improvement. This group was the only one that pinpointed the need for a process orientation and a holistic approach, including the entire value system, which they denoted *a lean hospital*. They mentioned all the enablers that were identified by the other focus groups. However, they diverged from the other two groups by placing their own role as internal consultants at the top of the list of important enablers. They also diverged by ranking management-anchoring and benefits’ motivating role above the need of a vision and perceived need for change among the staff.

### Staff

The staff recognized only some of the reviewed enablers. They did not mention a need for prior experience, competence or alignment, all of which are contextual enablers. Nor were clinical leadership or leadership by management, adaption or a holistic approach brought up. The staff emphasized the need for decentralized decision-making, clinic-anchoring and continuity of staff. They viewed lean as a meeting point, rather than a method of problem-solving. They shared the consultants’ emphasis on patient focus and bottom-up processes, but differed by stating that a conscious group composition was more important than internal consultants or management support. Assurances of sufficient and accessible resources were important for these stakeholders, as were credibility and trustworthiness concerning the lean initiative.

## Discussion

What happens when popular management ideas, like lean, travel into and within an organisation? We conclude that lean, being introduced by the management, taught and communicated by the internal consultants, and used in practical improvement work by the staff, is transformed and translated more than once on its way through the hospital.

There are numerous empirical studies on the adoption and implementation of management ideas, and also several attempts to theorize such processes. For example, a vast literature stream theorizes on and investigates organisations’ *absorptive capacity* and the role this plays in understanding variations in outcomes of adoption and implementation processes [32, 33]. There are also more rational and instrumental “how-to” theories, some of which argue for top-down implementation strategies [34, 35], while others consider bottom-up approaches as decisive for outcomes [36, 37]. At the other end of the spectrum are studies that consider management theories as fashions, and of organisations and their leaders as more or less dedicated fashion followers [38, 39]. Management fashions pass through populations of organisations as popularity curves of rapid upswings, followed by equally rapid downturns [40–42]. When conceptualized as fashion, management ideas are superficial phenomena that primarily affect organisations only on the surface, rather than impacting their core practices. Thus, a main assumption within this school of thought is that fashionable management ideas primarily lead to temporary discourse within organisations, which often remains decoupled from action [43–45].

This paper is based upon an alternative theory: that of organisational translation of practices and ideas [46–50]. Translation theory focuses on how ideas and various representations of practices travel in time and space. It contrasts with theories of diffusion, in which the ideas that spread resemble physical and hardly changeable objects [51]. Inherent in the diffusion approach is also the image of adopters as passive receivers, and of an active central broadcasting point that provides all of the energy to the dissemination process [52]. In contrast, translation theorists conceive management ideas as immaterial accounts that are transformed as they spread. The power behind the travel does not stem from one single, powerful source, but is created from the richness of the interpretations the idea triggers in each actor within a network [53].

Especially important for the interpretation of our study is the fact that the respondents were invited to identify local enablers of lean – that is, the content of the versions of lean they had developed and applied at the local level – and how these versions eventually related to the outcome of the intervention. This approach provides a window into the local translations of lean, in terms of the extent to which, how, and why lean is transformed. The conclusions are founded on the assumption that enablers represent a way of defining the lean version applied at the specific site under study.

### How lean is translated – the characterizing principles

Lean is, as indicated by our results, transformed at the hospital level. What characterizes local versions of lean? When the respondents were asked to identify the most important enablers for success, two-thirds was local, not identified in the literature review we conducted, which constitute the basis for this study. Among these were structural support from the management, palpable measures, a bottom-up approach, credibility, realism and patience. The top-three enablers for quality improvement identified in the review and supported locally were patient focus, teamwork, and a vision characterized by targets of urgency and direction. When it comes to what attributions make a considerable difference, according to the local stakeholders, local situational ones like credibility, bottom-up and problem, not method focus, dominates. This confirms previous research which has stated that lean interventions are social, complex and inherently context-dependent [8, 14, 54], and that local interpretation manifests in local versions of lean [55].

Based on the analysis of the enablers, we constructed three broad and intertwined guiding principles that characterize how local stakeholders translate lean. These three principles are the practical, the pragmatic and the sceptical way of handling lean.

#### The practical principle

The local version of lean is characterized as practical because it stresses preparation, process orientation, automatic data feedback to staff, and structural support from management. Lean is defined as a priority-setting tool that forces the organisation to rank activities according to their importance. When one specific quality improvement approach is chosen, the organisation must stick with it. In order to ensure continuous improvement, one single structure for monitoring, including a watchdog, is recommended.

#### The pragmatic principle

Lean is emphasized as a meeting place for problem solving, rather than a quality improvement method per se. For this reason, success depends on sufficient, but flexible, participation, and time and resources must be added when needed. A few palpable measures concerning professional issues and limited work processes will promote quick results and a smooth transition to everyday routine. Stakeholders state that an advantage of lean is that it is simple, flexible and less resource-demanding than other improvement tools. It represents a toolbox to pick from, quite accessible and straightforward.

#### The sceptical principle

Local stakeholders pay attention to the perceived need for change as a prerequisite for success. Lean must be comprehended as credible and trustworthy, and not as camouflage for dismissals and cuts. The outcomes should be evidence-based and compatible with professional values, without threatening the autonomy of professionals. A certain group composition that recognizes discord, includes critics and “owners” of the work processes at stake, and yet avoids enthusiasts, is suggested. Further on, the interventions must be results of a bottom-up approach that includes voluntariness and work-floor engagement. In addition, changes should be anchored in management, department and staff, facilitated by internal consultants recruited locally at the hospital. The improvement work should demonstrate realism and patience.

### The logics of lean translation

By separating the enablers identified by the stakeholders, local versions of lean can be vaguely discerned. If the enablers identified in the literature review truly mirror lean in healthcare, then only the consultants can be said to have stayed true to lean, as they shared all the reviewed enablers. Management shared the most, except for benefits as a motivation, a need for communication and feedback, and a holistic lean approach. The staff noted fewer known enablers, leaving out a need for clinical and management leadership, prior experience, lean competence, alignment to overall goals, a holistic approach and adaption. Only the consultants mentioned the need for a holistic *lean-hospital* mindset, and only the management saw the advantage of prior experience. The consultants and the management agreed on many issues, believing that lean is less resource-demanding than other quality improvement approaches, among other things. The staff and the management only had a few enablers in common. In the following, we outline and discuss three interrelated logics of the local translation of lean, which is believed to lead to the observed transformation.

#### “Whisper down the lane” – translation as a funnel

Translation can be understood as a multilayered process in which different parts of the organisation change the idea for their own use. The translation process functions as a funnel, or like the game “whisper down the lane,” where the work-floor version of lean diverges from the original idea. The local actors translate the idea into a world they know, based on appropriateness and sense-making [49].

Some impediments are rather obvious when it comes to the idea of lean’s travel within healthcare: the distinction between producing cars and giving care [56], the profound gap between evidence-based medicine and quality improvement storytelling [14], and the varying ability of hospitals to identify an idea, assimilate it, and exploit it to fulfil their own needs [33]. Szulanski [57] named these impediments as an arduous relationship, causal ambiguity and lack of absorptive capacity.

One important observation from the focus groups, in addition to the enablers the staff mentioned, was those that they did not mention. Classical enablers for quality improvement by lean were left out, such as the need for competence, alignment, adaption, process-orientation, and physicians and management engagement. The work-floor staff emphasized a belief in lean as a possible means for patient-directed problem solving, more than a method of quality improvement per se.

#### “Washed out” – copying the tools, leaving the philosophy out

A pragmatic way of implementing lean involve copying the tools, rather than the underlying philosophical elements [58]. Lillrank’s conceptual model for the transfer of management ideas is based on the observation that the greater the cultural and social distance, the more the output of a transfer process differs from the input [55]. Tools have a low level of abstractions, and are easy to transfer, while the lean philosophy requires a higher level of abstraction, and is thereby more demanding to implement.

Liker and Kaisha [25] state that most attempts to implement lean have been fairly superficial, because most organisations do not recognize that lean is an entire system that not only consists of tools, but also entails continuous learning, respect for people, and a long-term philosophy. Only the consultants in our study believed that a holistic approach and a *lean hospital* promote change. During its travel within the hospital, lean tools were adopted while the philosophy-part was washed out. The fact that, in the focus groups, the management emphasized lean as a less resource-demanding toolbox to pick from, and the perception of lean as a functional meeting place supports this assumption. This is in accordance with other studies [12, 54].

A statement from one of the respondents in the survey illustrates the problem with the logic of washing-out lean: *“(there is) a danger of (creating) a one-sided focus on process leaving out the corresponding focus on change in structure (restructuring) and change management. The consequence may be that the projects are restricted by meeting resistance in the established structures.”*

#### “Introductory sale” – conscious sell-in of the least controversial parts of lean

Manufacturing myths, a new vocabulary, differences in skills, professional or functional silos, hierarchy and resistance to change are among the barriers to lean in healthcare [59]. These barriers, which are caused by cultural and social distance may delay lean implementation in hospitals [54, 60]. Morris and Lancaster claim that management ideas have to be “boiled down” to be adaptable in a local setting [61]. Successful implementation of lean depends on effective adaption, and this in turn depends on translation [56].

Lean often leads to resistance, as do other industrial concepts and models of management [12, 54]. At the case hospital, the management relabelled lean as *patient pathway work*, perhaps as an attempt to weaken the coupling with industry and production, and thereby manage to reduce the anticipated resistance and *lean is mean* attitude reported elsewhere [12]. The internal consultants and the staff were told that quality improvements for the patients were the primary goal of lean, and that lean would not be used for economical savings or dismissals. In this way, the organisation left out the most controversial parts of lean in order to avoid resistance and to secure successful sell-in of the new idea [56].

A statement from one of the respondents in the survey may underline the logics of “sell-in:” *“With a declared patient-focus, it is also a paradox that the medical evidence-based literature is almost absent in lean. It is also strange how easily the ‘new’ terminology is adopted by management as matter of course, and then repeated – in constantly wider circles like ripples in water – without any knowledge of what we in fact know or do not know based on years of, often bitter, experience, and patient research. Lean may be an efficient management tool, but the terminology are and will always be out of place when employed for health services that are founded on a deeper and more nuanced value-base.”*

### Translation makes a difference

This study indicates that translation of lean makes a difference, specifically in two interrelated ways. First, the study illustrates the transformative power of translation [50, 62]. The local translation of lean at different levels and units of the organisation leads to a transformation of lean into various local versions within the organisation. Due to different translation processes, the work-floor versions of lean diverge from the original idea. They may also differ from each other. While some versions are slightly modified compared to the original idea, for instance by adding or toning down some elements, other versions are more radically transformed throughout the local translation processes [63, 64]. This mechanism throws light on the problems of measuring effects of lean interventions. Bluntly, lean is not lean, but more often numerous materialized versions of the idea; that may have, in methodological terms, various causes when it comes to measuring and comparing effects of lean interventions. It may be hard to account for these different versions in effect studies. In fact, we believe that translation makes a considerable contribution towards explaining the lack of evidence for lean; that is, the immaturity of the research field [12, 14, 65].

Second, there are reasons to believe that the ways in which translations are performed can be decisive for outcomes [47, 66]. Some translations may lead to successful lean interventions, while others cause the interventions to fail. Outcomes may depend on the extent to which, and in what way, lean is tailored to meet local needs. Thus, future research should focus on the relations between local translation processes and the effects of the interventions. In doing so, researchers should closely study, for example, the decisions that local actors make when translating lean, and reveal how they, in practice, balance two main concerns: on the one hand, concern for adapting lean to fit the local context and needs, and on the other hand, concern for staying true to lean, and making sure that the core elements of the concept are not washed out when tailoring it to new contexts.

### Strengths and weaknesses of the study

The focus on enablers was chosen for theoretical, as well as for analytical reasons. As a contribution to cure the lack of evidence for lean success, there is a growing body of literature on lean barriers and enablers [54, 67]. Observing that barriers often reflect a lack of enablers [59], we chose to focus on the latter. Enablers comprise the context and utilization, the content and outcomes of lean interventions, illuminating conditional attributions of the implementation process, i.e. the prevailing version of lean at the specific site.

A mixed-methods approach seemed best suited to explore the dissemination of lean within hospitals [68, 69]. Focus groups were used for exploring the stakeholders experience and attitudes towards lean. Group dynamics contributed to a more thorough exploration, taking the research in new and unexpected directions [69]. We argue that the design of three homogeneous focus groups encouraged a wider range of data to be detected, as well as it helped identifying group norms and social processes within those groups. By separating the three groups we were able to differentiate between three levels of the organisation; top-management, intermediate level, and work floor. In addition, we reduced the possibility for hierarchy to affect the data.

We suspected that the list of 44 broadly defined enablers lacked some clarity, and thereby were insufficient to guide policymakers how to achieve sustainable change [20]. The whole research field is characterized by these limitations [70, 71]. Some of this vagueness could be reduced by adding some specification of quantity to the dataset, i.e. how many, and who, hold which enablers as important. Given the fact that it is not appropriate to give percentages or frequency counts of focus group data [29], we decided to complement the data with a questionnaire, making it possible to state the relative importance of different enablers.

A relatively low response rate (49 pct.) may contribute to uncertainty about the results caused by sampling bias. The sample was representative regarding occupation and hierarchical level, but there may still be some unobserved imbalance in the sample.

By combining focus group interviews and the succeeding questionnaire, it was possible to cross-check reliability. And, by examination of whether the enablers identified in the literature review were retrieved at the hospital, we tested the validity of the former study. The findings from the two separate studies were anticipated to reinforce each other in a reciprocal manner.

Although the findings are based on a study at one single hospital, similar processes of translation can be expected at other sites, though they may result from other enablers than those identified here. The description and awareness of translation processes are relevant for organisations in general.

## Conclusions

Ideas travel, and so do quality improvement ideas. Lean management travelled all the way from Toyota in Japan to a university hospital in Norway. This study concerns the travel of lean through a hospital, from the top management, via internal consultants, to the work-floor staff, based on the assumption that local translation plays a key part in lean interventions.

The translation processes at the hospital were characterized by the practical, the pragmatic, and the sceptical principle, paying attention to the need of credibility, anchoring, realism and patience in lean interventions. Local attributions like these were the most important for local improvements. On its way through the hospital the idea of lean was translated, so that it eventually represented something different to the staff than it did to the top management that introduced it. The idea of lean was partly washed out, or edited, by management during their sell-in, and partly lost in translation via a funnel effect. We claim that translation is a considerable part of the explanation for why it is so hard to find proof of lean efficiency, and for the varying outcomes of lean interventions within and among hospitals.

The crux of lean-based quality improvement seems to be to capture the right balance between two main concerns: tailoring lean to local needs, and at the same time staying true to lean as a philosophy for change.

## Additional files

Additional file 1: Table S1. Study sample and data collection method. (DOCX 10 kb)
Additional file 2: Table S2. The local enablers. (DOCX 12 kb)
Additional file 3: Table S3. Reviewed and local enablers identified by the focus groups. (DOCX 13 kb)
Additional file 4: Tables S4-S7. The three most preferred enablers by management, consultants and staff in focus groups, percent per part of the intervention. (DOCX 13 kb)

## References

[1] Mazzocato P, Savage C, Brommels M, Aronsson H, Thor J (2010). Lean thinking in healthcare: a realist review of the literature. Qual Saf Health Care.

[2] Aherne J, Whelton J (2010). Applying lean in healthcare: a collection of international case studies.

[3] Kollberg B, Dahlgaard JJ, Brehmer PO (2007). Measuring lean initiatives in health care services: Issues and findings. Int J Product Perform Manag.

[4] Womack JP, Jones DT, Roos D. The Machine That Changed the World: the Story of Lean Production—Toyota's Secret Weapon in the Global Car Wars That Is Revolutionizing World Industry. New edn. In*.*: London: Simon & Schuster; 2007.

[5] Walshe K, Freeman T (2002). Effectiveness of quality improvement: learning from evaluations. Qual Saf Health Care.

[6] Walshe K (2007). Understanding what works—and why—in quality improvement: the need for theory-driven evaluation. Int J Qual Health Care.

[7] Plsek PE, Greenhalgh T (2001). The challenge of complexity in health care. BMJ.

[8] Davidoff F (2011). Systems of service: reflections on the moral foundations of improvement. BMJ Qual Saf.

[9] Pawson R, Greenhalgh T, Harvey G, Walshe K (2005). Realist review–a new method of systematic review designed for complex policy interventions. J Health Serv Res Policy.

[10] Shortell SM (1998). Assessing the Implementation and Impact of Clinical Quality Improvement Efforts: Abstract, Executive Summary and Final Report.

[11] Øvretveit J, Gustafson D (2002). Evaluation of quality improvement programmes. Qual Saf Health Care.

[12] Joosten T, Bongers I, Janssen R (2009). Application of lean thinking to health care: issues and observations. Int J Qual Health Care.

[13] Alexander JA, Hearld LR (2009). What Can We Learn From Quality Improvement Research? A Critical Review of Research Methods.

[14] Young T, McClean S (2008). A critical look at lean thinking in healthcare. Qual Saf Health Care.

[15] McIntosh B, Sheppy B, Cohen I (2014). Illusion or delusion-Lean management in the health sector. Int J Health Care Qual Assur.

[16] Andersen H, Røvik KA, Ingebrigtsen T (2014). Lean thinking in hospitals: is there a cure for the absence of evidence? A systematic review of reviews. BMJ Open.

[17] DIXON-WOODS M, Bosk CL, Aveling EL, Goeschel CA, Pronovost PJ (2011). Explaining Michigan: developing an ex post theory of a quality improvement program. Milbank Q.

[18] Yousri T, Khan Z, Chakrabarti D, Fernandes R, Wahab K (2011). Lean thinking: Can it improve the outcome of fracture neck of femur patients in a district general hospital?. Injury.

[19] Van Vliet EJ, Bredenhoff E, Sermeus W, Kop LM, Sol JC, Van Harten WH (2011). Exploring the relation between process design and efficiency in high-volume cataract pathways from a lean thinking perspective. Int J Qual Health Care.

[20] Bate P, Mendel P, Robert GB (2008). Organizing for quality: the improvement journeys of leading hospitals in Europe and the United States.

[21] Vos L, Chalmers SE, Dückers MLA, Groenewegen PP, Wagner C, Van Merode GG (2011). Towards an organisation-wide process-oriented organisation of care: A literature review. Implement Sci.

[22] Pedersen ERG, Huniche M (2011). Negotiating lean: the fluidity and solidity of new management technologies in the Danish public sector. Int J Product Perform Manag.

[23] Ingebrigtsen T, Lind M, Krogh T, Lægland J, Andersen H, Nerskogen E (2012). Merging of three hospitals into one university hospital. Tidsskr Nor Laegeforen.

[24] Womack JP, Jones DT (2010). Lean thinking: banish waste and create wealth in your corporation.

[25] Liker JK, Kaisha TJKK (2004). The Toyota way: 14 management principles from the world's greatest manufacturer.

[26] Bergseth HP, & Fossem, T. Evaluering av arbeidet med pasientforløp i UNN i perioden 2008–2012. In*.* Tromsoe, Norway: University hospital of North Norway; 2012: 22.

[27] Gremler DD (2004). The critical incident technique in service research. J Serv Res.

[28] Hart C (1998). Doing a literature review.

[29] Hinchcliff R, Greenfield D, Westbrook J, Pawsey M, Mumford V, Braithwaite J (2013). Stakeholder perspectives on implementing accreditation programs: a qualitative study of enabling factors. BMC Health Serv Res.

[30] Powell A, Rushmer R, Davies H (2008). A systematic narrative review of quality improvement models in health care.

[31] Kaplan HC, Provost LP, Froehle CM, Margolis PA (2012). The Model for Understanding Success in Quality (MUSIQ): building a theory of context in healthcare quality improvement. BMJ Qual Saf.

[32] Cohen WM, Levinthal DA (1990). Absorptive capacity: a new perspective on learning and innovation. Adm Sci Q.

[33] Lane PJ, Koka BR, Pathak S (2006). THE REIFICATION OF ABSORPTIVE CAPACITY: A CRITICAL REVIEW AND REJUVENATION OF THE CONSTRUCT. Acad Manag Rev.

[34] Fixsen DL, Blase KA, Naoom SF, Wallace F (2009). Core implementation components. Res Soc Work Pract.

[35] Sabatier PA (1986). Top-down and bottom-up approaches to implementation research: a critical analysis and suggested synthesis. J Publ Pol.

[36] Hjern B, Porter DO (1981). Implementation structures: a new unit of administrative analysis. Organ Stud.

[37] Long E, Franklin AL (2004). The paradox of implementing the government performance and results act: top‐down direction for bottom‐up implementation. Public Adm Rev.

[38] Abrahamson E (1996). Management fashion. Acad Manag Rev.

[39] Abrahamson E (1991). Managerial fads and fashions: the diffusion and rejection of innovations. Acad Manag Rev.

[40] Abrahamson E, Fairchild G (1999). Management fashion: lifecycles, triggers, and collective learning processes. Adm Sci Q.

[41] Pascale RT (1990). Managing on the edge: How the smartest companies use conflict to stay ahead.

[42] Walshe K (2009). Pseudoinnovation: the development and spread of healthcare quality improvement methodologies. Int J Qual Health Care.

[43] Cox JW, Minahan S (2006). Organizational decoration a new metaphor for organization development. J Appl Behav Sci.

[44] Ten Bos R (2000). Fashion and utopia in management thinking.

[45] Røvik KA (2011). From fashion to virus: an alternative theory of organizations’ handling of management ideas. Organ Stud.

[46] Czarniawska-Joerges B, Sevón G (2005). Global ideas: how ideas, objects and practices travel in a global economy.

[47] Czarniawska B, Sevón G (1996). Translating organizational change.

[48] Latour B (1986). The powers of association.

[49] Czarniawska B, Joerges B (1996). Travels of ideas.

[50] Nielsen JA, Mathiassen L, Newell S (2014). Theorization and translation in Information Technology institutionalization: evidence from Danish home care. MIS Q.

[51] Rogers EM, Cartano DG (1962). Living research methods of measuring opinion leadership. Publ Opin Q.

[52] Powell WW, Gammal DL, Simard C (2005). Close encounters: the circulation and reception of managerial practices in the San Francisco Bay area nonprofit community.

[53] Brown SD (2002). Michel Serres. Theor Cult Soc.

[54] Radnor ZJ, Holweg M, Waring J (2012). Lean in healthcare: The unfilled promise?. Soc Sci Med.

[55] Lillrank P (1995). The transfer of management innovations from Japan. Organ Stud.

[56] Waring JJ, Bishop S (2010). Lean healthcare: rhetoric, ritual and resistance. Soc Sci Med.

[57] Szulanski G (1996). Exploring internal stickiness: impediments to the transfer of best practice within the firm. Strateg Manag J.

[58] Cole RE (1998). Learning from the quality movement: what did and didn't happen and why?. Calif Manag Rev.

[59] de Souza LB, Pidd M (2011). Exploring the barriers to lean health care implementation. Publ Money Manag.

[60] Kostova T (1999). Transnational transfer of strategic organizational practices: a contextual perspective. Acad Manag Rev.

[61] Morris T, Lancaster Z (2006). Translating management ideas. Organ Stud.

[62] Barley SR (1986). Technology as an occasion for structuring: evidence from observations of CT scanners and the social order of radiology departments. Adm Sci Q.

[63] Westney DE, Westney DE (1987). Imitation and innovation: the transfer of Western organizational patterns to Meiji Japan.

[64] Wæraas A, Sataøen HL. Trapped in conformity? Translating reputation management into practice. Scandinavian Journal of Management 2013. http://dx.doi.org/10.1016/j.scaman.2013.05.002.

[65] Shortell SM, Bennett CL, Byck GR (1998). Assessing the impact of continuous quality improvement on clinical practice: What it will take to accelerate progress. Milbank Q.

[66] Trägårdh B, Lindberg K (2004). Curing a meagre health care system by lean methods—translating ‘chains of care’in the Swedish health care sector. Int J Health Plann Manag.

[67] Rycroft-Malone J, Kitson A, Harvey G, McCormack B, Seers K, Titchen A (2002). Ingredients for change: revisiting a conceptual framework. Qual Saf Health Care.

[68] Patton MQ (2005). Qualitative research.

[69] Kitzinger J (1995). Qualitative research. Introducing focus groups. BMJ.

[70] Øvretveit J (2011). Understanding the conditions for improvement: research to discover which context influences affect improvement success. BMJ Qual Saf.

[71] Al-Balushi S, Sohal A, Singh PJ, Al Hajri A, Al Farsi Y, Al Abri R (2014). Readiness factors for lean implementation in healthcare settings–a literature review. J Health Organ Manag.

