# Supplementary material for: Lost in translation: a case-study of the travel of lean thinking in a hospital
Source: BMC Health Serv Res. 2015 Sep 21;15:401. doi: 10.1186/s12913-015-1081-z (PMC4578238; doi:10.1186/s12913-015-1081-z)
Supplement: Additional file 1: Table S1. — Study sample and data collection method. (DOCX 10 kb) [file 12913_2015_1081_MOESM1_ESM.docx]

**Additional file 1**

**Table A1: Study sample and data collection method**

| Stakeholder group | Focus group interview | Questionnaire | Total number |
| --- | --- | --- | --- |
| Steering group leaders/members | 8 | 40 | 48 |
| Internal consultants | 14 | 23 | 37 |
| Implementation staff | 11 | 102 | 113 |
| Total number | 33 | 165 | 198 |
