# Supplementary material for: Lost in translation: a case-study of the travel of lean thinking in a hospital
Source: BMC Health Serv Res. 2015 Sep 21;15:401. doi: 10.1186/s12913-015-1081-z (PMC4578238; doi:10.1186/s12913-015-1081-z)
Supplement: Additional file 2: Table S2. — The local enablers. (DOCX 12 kb) [file 12913_2015_1081_MOESM2_ESM.docx]

**Additional file 2**

**Table A2: The local enablers**

| Enabler | Description |
| --- | --- |
| Preparation | Preparatory work, baseline established |
| Need for change | Perceived need, potential for improvement |
| Anchoring in management, department or staff | “lean management” |
| Management structure support | Organizational structural support, coordination and continuity in management and staff |
| Bottom-up | Improvement suggestions from floor, voluntariness due to initiative |
| Dedication to lean | Stick with lean and stay true to the method |
| Process orientation | work processes in focus |
| Priority setting tool | Enforce priorities |
| Visual and simple, less resource demanding | Tool box to pick from, spot check, not science |
| Credibility | No bragging, trustworthiness, no camouflaged dismissals and cuts |
| Internal consultants | Project management skills, mentors and network |
| Group composition | Include critics, recognize discord. “Owners” participate, roles clarification. Avoid enthusiasts |
| Operational | Professional issues at stake, intensive small cases |
| Sufficient participation | Sufficient, but flexible. Add resources and time when necessary |
| Problem, not method focus | Lean as a meeting place |
| Compatible to professional values | Outcomes; Not threaten autonomy, evidence based |
| Data feedback | Information to staff, high level analysis, automatic data collection |
| Smooth transition | From project to every day routine. Agreements commit |
| Realism and patience | Distinct mandate, demarcation, smaller projects. Adjustment possible |
| Few, palpable measures | Concrete, quick results and visual success-stories |
| Follow-up structure | One standard established, Focus and progress, watch dog ask for results |
