# Supplementary material for: Lost in translation: a case-study of the travel of lean thinking in a hospital
Source: BMC Health Serv Res. 2015 Sep 21;15:401. doi: 10.1186/s12913-015-1081-z (PMC4578238; doi:10.1186/s12913-015-1081-z)
Supplement: Additional file 3: Table S3. — Reviewed and local enablers identified by the focus groups. (DOCX 13 kb) [file 12913_2015_1081_MOESM3_ESM.docx]

**Additional file 3**

**Table A3: Reviewed and local enablers identified by the focus groups**

|  | Enabler | Management | Internal consultants | Staff |
| --- | --- | --- | --- | --- |
| Reviewed enablers | Experience | X |  |  |
|  | Belief |  | X | X |
|  | IT-systems | X | X | X |
|  | Competence | X | X |  |
|  | Alignment | X | X |  |
|  | Vision | X | X | X |
|  | External support* |  |  |  |
|  | Adaption | X | X |  |
|  | Customer focus | X | X | X |
|  | Training | X | X | X |
|  | Resources | X | X | X |
|  | Accurate data | X | X | X |
|  | Teamwork | X | X | X |
|  | Administrative support | X | X | X |
|  | Physicians | X | X |  |
|  | Management | X | X |  |
|  | Staff involvement | X | X | X |
|  | Supportive culture | X | X | X |
|  | Communication |  | X | X |
|  | Holistic approach |  | X |  |
|  | Continuous improvement | X | X | X |
|  | Measurement | X | X | X |
|  | System-wide scope | X | X | X |
| Local enablers | Preparation | X | X | X |
|  | Need for change | X | X |  |
|  | Anchoring in management, department or staff | X | X | X |
|  | Management structure support | X | X | X |
|  | Bottom-up | X | X | X |
|  | Dedication to lean | X | X |  |
|  | Process orientation |  | X |  |
|  | Priority setting tool | X | X | X |
|  | Visual and simple, less resource demanding | X | X |  |
|  | Credibility |  | X | X |
|  | Internal consultants | X | X | X |
|  | Group composition | X | X | X |
|  | Operational | X | X | X |
|  | Sufficient participation | X | X | X |
|  | Problem, not method focus | X | X | X |
|  | Compatible to professional values |  | X | X |
|  | Data feedback | X | X | X |
|  | Smooth transition | X | X | X |
|  | Realism and patience | X | X | X |
|  | Few, palpable measures | X | X | X |
|  | Follow-up structure | X | X | X |

* Enabler only identified in the review
