# Supplementary material for: Lost in translation: a case-study of the travel of lean thinking in a hospital
Source: BMC Health Serv Res. 2015 Sep 21;15:401. doi: 10.1186/s12913-015-1081-z (PMC4578238; doi:10.1186/s12913-015-1081-z)
Supplement: Additional file 4: Tables S4-S7. — The three most preferred enablers by management, consultants and staff in focus groups, percent per part of the intervention. (DOCX 13 kb) [file 12913_2015_1081_MOESM4_ESM.docx]

**Additional file 4**

**Table A4-7: The three most preferred enablers by management, consultants and staff in focus groups, percent per part of the intervention**

| **Context** | Management structure | Vision | Need for change | Anchoring in management | Belief in benefits |
| --- | --- | --- | --- | --- | --- |
| Total | 59 | 48 | 36 | 36 | 17 |
| Management | 55 | 60 | 40 |  |  |
| Staff | 60 | 47 | 39 |  |  |
| Consultants | 61 |  |  | 44 | 39 |

| **Content** | Costumer focus | Bottom-up | Problem, not method focus |
| --- | --- | --- | --- |
| Total | 58 | 49 | 47 |
| Management | 48 | 50 | 58 |
| Staff | 59 | 48 | 43 |
| Consultants | 61 | 44 | 44 |

| **Application** | Teamwork | Credibility | Internal consultants | Group composition | Management |
| --- | --- | --- | --- | --- | --- |
| Total | 61 | 34 | 34 | 33 | 19 |
| Management | 58 |  | 38 |  | 38 |
| Staff | 63 | 34 |  | 32 |  |
| Consultants | 48 | 30 | 61 |  |  |

| **Outcomes** | Few, palpable measures | Realism and patience | Holistic approach | Smooth transition |
| --- | --- | --- | --- | --- |
| Total | 66 | 45 | 43 | 29 |
| Management | 68 |  | 40 | 48 |
| Staff | 65 | 47 | 47 |  |
| Consultants | 61 | 52 | 30 | 30 |
